# Keeping a lid on nodal: transcriptional and translational repression of nodal signalling

**DOI:** 10.1098/rsob.150200

**Published:** 2016-01-20

**Authors:** Karuna Sampath, Elizabeth J. Robertson

**Affiliations:** 1 Division of Biomedical Sciences, Warwick Medical School, University of Warwick, Coventry CV4 7AJ, UK; 2 Sir William Dunn School of Pathology, Oxford University, Oxford OX1 3RE, UK

**Keywords:** nodal signalling, transcription, translation, localization, repression, development

## Abstract

Nodal is an evolutionarily conserved member of the transforming growth factor-*β* (TGF-β) superfamily of secreted signalling factors. Nodal factors are known to play key roles in embryonic development and asymmetry in a variety of organisms ranging from hydra and sea urchins to fish, mice and humans. In addition to embryonic patterning, Nodal signalling is required for maintenance of human embryonic stem cell pluripotency and mis-regulated Nodal signalling has been found associated with tumour metastases. Therefore, precise and timely regulation of this pathway is essential. Here, we discuss recent evidence from sea urchins, frogs, fish, mice and humans that show a role for transcriptional and translational repression of Nodal signalling during early development.

## 1. Introduction

Nodal is a member of the TGF-β superfamily of secreted signalling factors. Identified first via a retroviral insertion mutation that affected development of mouse embryos homozygous for the insertion, the RNA was found to be expressed in the node, a group of cells in the early mouse embryo that arise from the anterior-most part of the primitive streak, and which gives rise to the notochord, an axial midline structure that plays crucial roles in embryonic patterning [1,2]. Nodal factors play important roles in axis formation and germ-layer specification during embryonic development in sea urchins, ascidians, frogs, fish, chicks and mammals [3–9]. Nodal homologues in gastropod molluscs regulate the chirality of snail shells [4], and a recent study in hydra showed a role for nodal in establishing axial asymmetry and lateral branching in bilaterians [10]. In the indirect developing hemichordate, *Ptychodera flava*, Nodal is required for formation of larval mesoderm and specification of ventral cell fates [11]. Thus, Nodal is an evolutionarily conserved factor with key roles in metazoan development. Nodal signalling is also important for maintaining human embryonic stem cell (hESC) pluripotency [12,13]. Mis-regulation of Nodal signalling has been found associated with tumour metastases, although the mechanisms are unclear [14]. Therefore, precise regulation of Nodal signalling is required for normal development and for maintaining homeostasis.

Nodal signalling is regulated at the transcriptional level by factors such as DRAP1, FoxH1, RBPjk and Oct4 [15–20]. Signal transduction occurs by binding of the Nodal ligands to the receptor complex, leading to activation of downstream Smad effectors [21,22]. Feedback regulation of Nodal signalling by the Lefty inhibitors has been shown to be important in several organisms [23–28]. A role for miRNAs in post-transcriptional regulation of Nodal signalling has been identified in *Xenopus* and zebrafish embryos, and in trophoblast cells of first trimester human placental explants [29–32]. Nodal proteins are also influenced by secretion, endocytosis, post-translational modifications and processing of the ligands [7,8,33–37]. The regulation of Nodal signalling by these modes has been reviewed extensively [7,8,22,38,39], and will not be covered here. Here, we discuss recent evidence that shows a role for transcriptional and translational repression in controlling Nodal signalling during the earliest events of embryogenesis.

## 2. Transcriptional repression of the nodal pathway

Transcription factor complexes play key roles in cell-fate specification during development. The Nodal pathway acts through the transcription factors Smad2/3 to regulate many aspects of development and differentiation in a variety of organisms (reviewed in [22]). Targets of Smad2/3 include the *nodal* genes themselves, as well as the *lefty* inhibitors of Nodal signalling. The forkhead domain transcription factor FoxH1 interacts with the Wnt/TCF/β-catenin pathway to activate *nodal* expression in early *Xenopus* embryos [40]. However, this interaction is thought to be independent of Smad2. A recent study showed that the basic helix-loop helix transcription factor E2a functions in repression of Nodal signalling [41]. By association with other cofactors, E2a can have widespread effects on transcriptional regulation of the genome. For instance, E2a acts as either a transcriptional activator or repressor in B cells, depending on its cofactors. By analysis of CHIP-seq and RNA-seq datasets generated from *Xenopus tropicalis* embryos depleted of E2a, Wills & Baker [41] found that E2a is not required for direct association of Smad2/3with chromatin during gastrulation. Rather, E2a positions Smad2/3 at the *lefty* genomic locus, and represses lefty transcription. Overexpression of E2a mRNA in early *Xenopus* embryos reduced *lefty* expression. E2a acts as a repressor in this context by altering Smad2/3 occupancy from *lefty* regulatory regions, and displacing Smad2/3 from a region associated with transcriptional activation. Intriguingly, E2a also appears to have role as a transcriptional activator of target genes such as *epha4* and *eomesodermin*. Smad2/3 is positioned at these loci, but transcription is repressed in the absence of E2a activity. The co-activator for E2a in transcriptional activation of these loci is not known.

Repression of *nodal* transcription plays an important role in establishing the dorsoventral axis and oral–aboral axes in sea urchin embryos [42]. In sea urchin blastulae, nodal is specifically expressed in a small group of cells that defines their ventral identity and acts as a dorsoventral organizing centre. In the dorsal ectoderm, the homeobox containing factor Hbox12 prevents activation of nodal transcription [42]. Injection of either hbox12 RNA or a chimeric hbox12-engrailed repressor RNA into zygotes led to attenuation of nodal transcript levels, and loss of bilateral symmetry. Although several consensus binding-sites for homeodomain-containing factors have been identified in the *nodal* promoter sequences [43], it is not known if Hbox12 directly represses *nodal* transcription in dorsal cells.

In the sea urchin apical neurogenic ectoderm, FoxQ2 together with the Nodal agonist Lefty, suppresses nodal expression [44]. FoxQ2 is normally restricted the animal plate, and increased or ectopic FoxQ2 expression leads to a block in *nodal* expression and disruption of oral–aboral polarity, leading to radialized embryos. Thus, FoxQ2 prevents premature activation of nodal in the ectoderm prior to vegetal signalling. In amphioxus embryos, foxq2 is expressed in cleavage stages prior to expression of nodal transcripts in foxq2-negative cells, raising the possibility of FoxQ2 regulation of Nodal in chordates [45]. It is not known if the vertebrate FoxQ2 orthologues repress nodal transcription in early embryos.

## 3. Translational repression of nodal signalling

### 3.1. Repression of maternal Sqt/nodal in zebrafish

In the zebrafish genome, there are three nodal-related genes: *cyclops*, *squint* and *southpaw*. Of these, *squint*/*nodal-related 1* transcript expression is detected in pre-blastula stage zebrafish embryos, whereas *cyclops*/*nodal-related 2* is expressed from late blastula stages in the blastoderm margin, in the axial mesendoderm during gastrulation, and subsequently in the left diencephalon and left lateral plate mesoderm (LPM) [46–49]. Expression of *southpaw*/*nodal-related 3* is observed in the left LPM and left diencephalon during late somitogenesis [50]. Among other core nodal pathway components, transcripts encoding the Nodal receptors, co-receptor One-eyed pinhead (oep), the downstream effectors Smad2 and Smad3, and FoxH1 are expressed both maternally and zygotically, whereas the Nodal inhibitors lefty1 and lefty2 are detected after the mid-blastula transition (MBT) in the early embryo [25,51–58].

**Figure 1.**
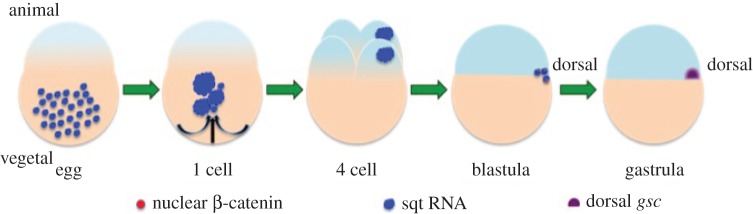
Schematic of maternal sqt/nodal RNA expression in early zebrafish embryos. Sqt RNA localizes by the four-cell stage in one or two cells, which later form dorsal progenitors, marked by nuclear β-catenin and *gsc* expression.

Whereas zygotic squint (sqt) RNA is expressed in the blastula margin, the dorsal yolk syncytial layer (YSL), and the dorsal organizer, the shield, maternal sqt transcripts are expressed in oocytes and eggs, where its spatial expression pattern is ubiquitous [9,46,49,59]. Upon egg activation and fertilization, there is a change in the uniform distribution of maternal sqt RNA. Aggregates of sqt RNA form, fuse and are transported to the blastoderm in a microtubule-dependent manner (figure 1) [46]. The RNA is then asymmetrically distributed in one or two adjacent cells by the four-cell stage. Localization of sqt RNA in early embryos does not correlate with the planes of early cell divisions or with the microtubule-organizing centre. However, the cells that acquire sqt RNA later become embryonic dorsal progenitors [47]. Thus, localization of maternal sqt RNA predicts the embryonic dorsal axis of zebrafish embryos by the four-cell stage, demonstrating the first visible molecular asymmetry in the embryonic blastoderm.

Maternal sqt RNA is detected in an unprocessed form in eggs, in that it lacks a polyA tail possessed by mRNAs that are ready to be translated. Thus, sqt RNA is among a cohort of maternal transcripts in zebrafish eggs that are present as non-polyadenylated RNAs and show delayed poly-adenylation at pre-MBT stages [60,61]. In *Drosophila* and *Xenopus* eggs, some maternal transcripts are de-adenylated during oogenesis, followed by re-polyadenylation after fertilization, particularly during blastula and gastrula stages [62–64]. This is likely to be a mechanism to prevent precocious translation of the maternal RNAs until the protein functions are required. In addition to being non-polyadenylated, maternal sqt is detected as unspliced pre-mRNA in early cleavage stage zebrafish embryos [61,65]. The presence of unprocessed sqt RNA in early embryos suggests that maternal sqt is not translated at these stages, and consequently Sqt/Nodal protein is not available to activate the signalling pathway.

Indeed, experiments to block the response to Nodal signalling by treatment of embryos with the ALK inhibitor drugs SB 431542 and SB 505124 at different times showed that the Nodal receptor complex is not required for signalling until late blastula stages. Experiments to rescue maternal zygotic one-eyed pinhead (MZ*oep*) mutant embryos by injections of oep mRNA at different times also support a requirement for the co-receptor only from late blastula stages [66–69]. Phosphorylation of Smad2, the downstream effector of Nodal signalling, is also observed only from late blastula stages [70]. Therefore, although maternal sqt/nodal RNA is expressed and localizes to dorsal progenitors at very early stages, Nodal signalling is not active in zebrafish embryos until the late blastula (3 hpf).

### 3.2. Regulation of maternal Sqt/nodal by Ybx1

**Figure 2.**
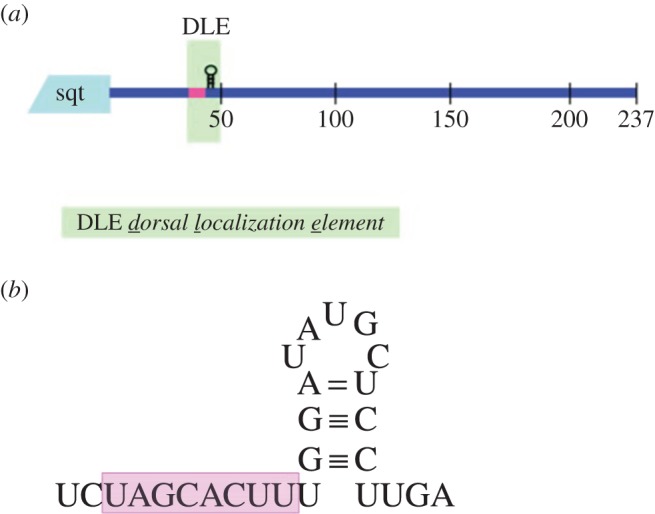
Schematic of sqt DLE sequence and structure motif. (*a*) Schematic of sqt exon 3 and the 3′-UTR, showing the dorsal localization element (DLE; green shading), which includes a single-stranded motif (pink box) and a short stem-loop structure. (*b*) The sequence of the single-stranded motif (pink highlighted region) and the stem-loop structure of the hairpin are shown.

How is Sqt/Nodal signalling kept inactive in early zebrafish embryos even though maternal sqt RNA is expressed in oocytes and eggs? RNA localization and translational control are key regulatory steps for many RNAs, and elements within the RNAs can control their translation, in a manner akin to *cis*-regulatory elements in DNA that function in transcriptional regulation. A key regulatory module in sqt RNA that functions in its localization and expression resides in its 3′-untranslated region (3′-UTR): the dorsal localization element (DLE) [71]. The DLE comprises a predicted single-stranded region that harbours an AGCAC sequence motif, followed by a stem-loop structure (figure 2). Nucleotide substitutions in the single-stranded region showed that the sequence of this motif is crucial for sqt localization to dorsal progenitors. The nucleotide sequence of the stem and the loop region is not critical, but the structure of the stem-loop region appears to be required, because mutations that disrupt base pairing in the stem region abolish sqt RNA localization to dorsal progenitors. Conversely, compensatory mutations that restore base pairing in the stem region (regardless of nucleotide sequence) restore dorsal localization. Therefore, the DLE comprises both sequence and structural features [71].

Stem-loop structures in RNAs are sites that can be bound by protein complexes that regulate the RNA. Indeed, the sqt DLE is recognized and bound by the conserved nucleic-acid-binding protein, Y box-binding protein 1 (Ybx1) [70]. Ybx1 or its mammalian orthologue, YB1, is a 50 kDa protein that belongs to the cold shock domain (CSD) family of proteins [72]. CSD proteins contain an evolutionary conserved sequence near the N-terminus, the ‘CSD’, which was first identified in Cold shock protein A (CspA) from *Escherichia* *coli* [73]. Bacterial CSD proteins are important for adaptation to lower temperatures [74].

Members of this group include Lin-28 in *Caenorhabditis elegans*, FRGY1 in *Xenopus laevis*, YB1 in chicks, mice and humans, and MSY2 and MSY4 in mice [72,75–77]. The vertebrate Ybx1 homologues also share, in addition to the CSD, an alanine and proline rich N-terminus region, a dimerization domain, and a C-terminal domain that contains charged amino acid residues and encompasses a non-canonical nuclear localization signal. Ybx1 is an abundant protein with many functions. It is the major protein bound to mRNAs in rabbit reticulocytes. Mammalian YB-1 is a nucleic-acid-binding protein involved in translational regulation of genes such as *snail1*, which are associated with epithelial–mesenchymal transition (EMT) [78].

In *Drosophila melanogaster* and *Ciona intestinalis*, Ybx1 homologues are maternally deposited and associated with localized RNAs. For instance, Ypsilon schachtel (Yps) was found in the oskar RNA localization complex, and CiYB1 was observed in storage mRNP granules in ascidian oocytes [79,80]. The *Xenopus* Ybx1 protein, FRGY2, is a major component of storage mRNA particles in oocytes, and binds to an AACUAC sequence motif in RNA [75,81–83]. Ybx1 is a fairly abundant component of RNP complexes in neurons, where it is thought to function as an activity-dependent translational repressor of GluR2 and CaM1 RNAs [84,85]. Ybx1 has also been found associated with Fragile × Mental Retardation protein (FMRP) RNA granules. The association of mouse YB1/p50 with FMRP in neuronal mRNP particles is thought to be required for translational modulation [86]. In mice, mutations in YB1 lead to embryonic lethality and severe growth retardation [87].

**Figure 3.**
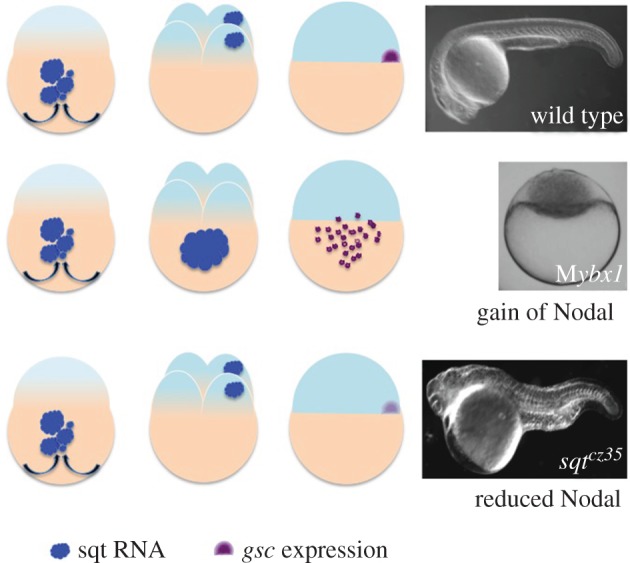
Schematic of zebrafish ybx1 and sqt/nodal mutant phenotypes. In *sqt* mutants *gsc* expression is initiated but not maintained, whereas in M*ybx1* mutants, sqt RNA is mis-localized and *gsc* is expressed prematurely in the expanded yolk syncytial layer (YSL). DIC images show the phenotypes of wild-type or mutant embryos.

**Figure 4.**
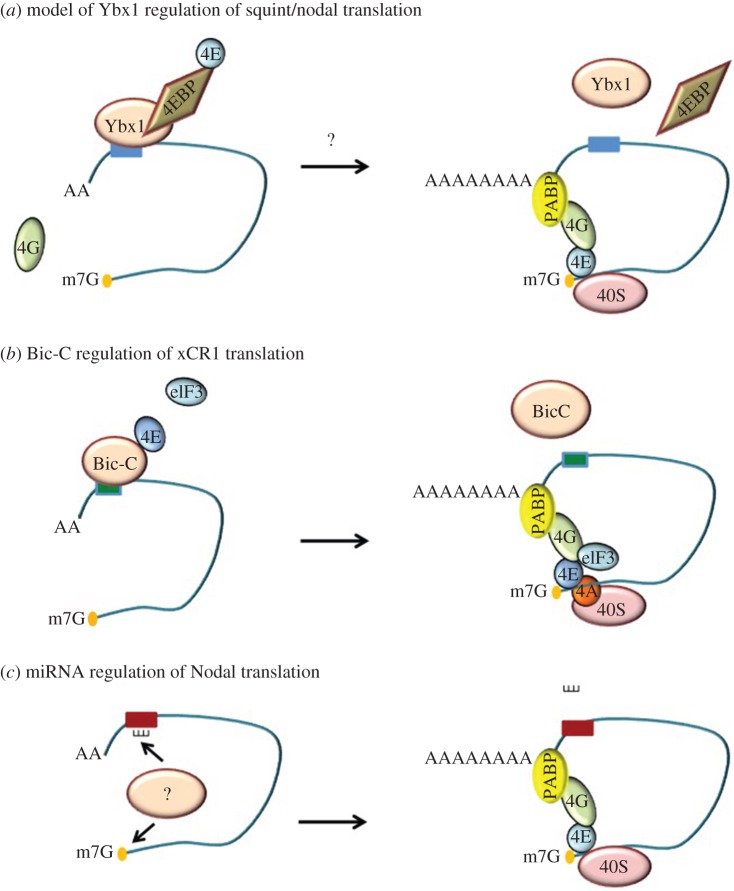
Model of translational repression of Nodal pathway components. (*a*) In early zebrafish embryos, Ybx1 represses squint/nodal translation by binding to the translation pre-initiation complex proteins and the squint DLE (solid blue box). Squint/Nodal translation is activated from late blastula stages by unknown factors. (*b*) In vegetal cells of frog embryos, Bic-C represses xCR1 translation by binding to the translation pre-initiation complex proteins and the xCR1 TCE (solid green box). In animal cap cells, xCR1 translation is activated in the absence of Bic-C. (*c*) miRNA-378a-5p (black comb) binds to the 3′-UTR (solid red box) of human Nodal RNA and unknown factors (?) to repress Nodal translation in the human placenta. In the absence of miRNA-378a-5p, nodal translation is activated.

In zebrafish, maternal Ybx1 protein is required for localization of maternal sqt RNA, and maternal effect mutants affecting *ybx1* lead to sqt RNA accumulation in the yolk (figure 3). In addition to affecting sqt localization, Ybx1 also has a role in regulation of sqt RNA processing. Maternal *ybx1* mutant embryos (M*ybx1*) show premature polyadenylation and splicing of sqt pre-mRNA [70]. Ybx1 binds to the m7G cap-binding protein eIF4E protein, and likely prevents the translation initiation complex from assembling on mRNAs (figure 4). Consequently, in M*ybx1* mutants, Sqt protein is translated prematurely, leading to precocious activation of Nodal signalling in the early zebrafish embryo. This leads to precocious specification and increased numbers of extra-embryonic YSL nuclei, and the blastoderm is depleted of cells, leading to failure to initiate gastrulation and lethality [70] (figure 3). Thus, maternal Ybx1 is a key regulator of maternal nodal and early development in zebrafish.

### 3.3. Restricted translation of the nodal cofactor Cripto in *Xenopus* embryos

The Cripto protein is an extracellular glycophosphatidylinositol (GPI)-linked membrane protein thought to function as a Nodal co-receptor. Cripto activity is required to establish a Nodal–Smad2 auto-regulatory loop in nodal signalling, and mutations in mouse Cripto that disrupt interactions with Nodal and Smad2 (Cripto^F78A^), result in embryos that manifest arrested gastrulation and impaired Nodal signalling [88]. In *Xenopus*, the Cripto-1 protein xCR1 is required for nodal signalling and anterior–posterior patterning [89–91]. Although xCR1 RNA is deposited maternally, the RNA has a short polyA tail of less than 20 nucleotides in oocytes and early embryos, and polyribosomes are only found associated with xCR1 RNA in embryos after the 32-cell stage. The length of the polyA tail increases during embryogenesis, coincident with polyribosome association with xCR1 RNA. These observations suggest that xCR1 is not translationally active in frog oocytes and early embryos. There are spatial differences in polyribosome association of xCR1, with enrichment observed in the animal pole. Moreover, an exogenously provided luciferase reporter fused with xCR1 3′-UTR sequences was translated efficiently in animal pole cells compared with vegetal cells. These findings suggest that the 3′-UTR of xCR1 is repressed in vegetal cells [92].

A specific region of the 3′-UTR functions in vegetal-cell specific translational repression of xCR1 [92]. The 3′-UTR-mediated translational repression of xCR1 in vegetal cells requires the 5′-cap and the translation initiation factors eIF4F and eIF3. The RNA-binding protein Bicaudal-C (Bic-C) has been shown to be restricted to vegetal cells and is associated with several maternal RNAs including xCR1 mRNA [93]. Luciferase reporters containing the 3′-UTR of xCR1 were repressed by Bic-C in ectopic repression assays in frog embryos, and Bic-C bound a 32-nt region of the xCR1 3′-UTR via its KH-domains, which are known to bind to RNA [94]. These experiments suggest that Bic-C spatially restricts translation of xCR1 in vegetal cells of early frog embryos (figure 4). In addition to xCR1, other components of the Nodal pathway such as Smad4 and Coco RNA have also been found to bind to Bic-C [93]. However, it is not known how Bic-C regulates signalling via these Nodal/TGF-β pathway molecules, or if Bic-C repression of Cripto is conserved in other organisms.

### 3.4. Nodal regulation in mouse embryos prior to and during implantation

In addition to its well-documented expression and functions in the epiblast, node and left LPM [22], nodal is also expressed in mouse embryos prior to implantation. Nodal RNA is detected in the blastocyst from E3.5 [95], and in the inner cell mass (ICM) and primitive endoderm (PrE) of the blastocyst by E4.5 [96]. In addition to Nodal, several components of the Nodal pathway are expressed prior to and during implantation of mouse embryos at E4.5. For instance, the Nodal co-receptor Cripto, the intracellular effectors of Nodal signalling Smad2 and Smad3, and the transcription factor FoxH1, are present in E3.5 mouse embryos. The Nodal antagonist Lefty1 is also expressed from E3.5 onwards. The precise function of early nodal expression in mouse embryos is unknown, but nodal deficient blastocysts develop normally and correctly specify all three primary cell populations namely the trophectoderm, ICM and primitive endoderm. Reporters for two regulatory regions in the Nodal locus show that the Wnt-dependent proximal epiblast enhancer (PEE) and asymmetric FoxH1-dependent enhancer (ASE) regions are responsible for expression prior to implantation [95]. Nodal signalling and FoxH1 are not required for activation of nodal at pre-implantation stages, whereas Wnt/β-catenin signalling appears to have an important role in maintaining Nodal transcription in the epiblast [97]. However, it is not known precisely which factors and mechanisms control expression of nodal or how Nodal signalling is repressed prior to implantation.

### 3.5. Nodal has essential roles in the early mouse embryo

Evidence for an early requirement for Nodal in induction of posterior mesodermal fates comes from studies of Nodal null embryos in which the epiblast differentiates to uniformly acquire neural character [98]. Molecular markers of pluripotency in the epiblast, such as the transcription factors Nanog, Oct4 and Foxd3, and the Nodal co-receptor Cripto, are rapidly downregulated in nodal^−/−^ mutant embryos [96]. Nanog mRNA, which is normally expressed at high levels in early blastocysts and at lower levels during egg cylinder formation (E5.0), is similarly barely expressed in nodal^−/−^ mutants. Thus, Nodal is required before E5.5 to maintain pluripotency in the epiblast [96]. In mouse stem cells, Nodal/Activin signalling is required for epiblast stem cell self-renewal, but not for ESCs [99,100]. Moreover, in embryo explant cultures, Nodal signalling is necessary and sufficient to maintain Oct4 expression in the epiblast. These reports support an essential function for Nodal/Smad2/3 signalling in maintenance of the pluripotent state of the early epiblast prior to gastrulation.

### 3.6. Regulation of nodal signalling in the human placenta

Recent work on human trophoblasts has revealed a role for Nodal signalling in human placental development [32]. Nodal inhibits trophoblast proliferation, invasion and migration, and induces apoptosis in placental explants. This activity of Nodal is mediated via the ALK 7 receptor [101,102]. Nodal expression in the placenta is suppressed by miRNA-378a-5p, which targets a region of the nodal 3′UTR. Stable transfection of miRNA-378a-5p in the human trophoblast cell line, HTR8/SV neo, decreases Nodal protein levels and represses expression of luciferase reporters harbouring Nodal 3′-UTR sequences, indicating that miRNA-378a-5p regulates Nodal protein translation. Mutations in the miRNA-378a-5p target site of the Nodal 3′-UTR can restore luciferase reporter expression. In placental explants, the outgrowth of extravillous cytotrophoblasts is enhanced by miRNA-378a-5p, and it has been reported that miRNA-378a-5p levels are reduced in preterm pregnancies of pre-eclamptic women [32]. These findings suggest that repression of Nodal protein translation by miRNA-378a-5p might play an important role in human placental development.

## 4. Concluding remarks and perspectives

These lines of evidence raise several interesting questions. What is the precise role of nodal in pre-implantation mammalian embryos? The nodal pathway is expressed and poised for activation prior to implantation, but there have been suggestions that to promote pluripotency and delay cell-fate decisions in the early mouse embryo Nodal signalling may need to be dampened prior to implantation [95]. Is translational regulation used in all the various contexts that the Nodal pathway is deployed? Even though there is no evidence that nodal RNA is localized in the early mammalian embryo, the 3′-UTR of mammalian nodals can localize heterologous reporters to dorsal progenitors in zebrafish embryos [47]. The DLE in zebrafish sqt/nodal, in addition to being required for localization of maternal sqt, also functions in repression of Nodal protein translation via Ybx1, and de-regulation of Nodal signalling leads to excess of the extra-embryonic YSL. This raises the possibility that the 3′-UTR of *nodal* harbours a conserved Ybx1-mediated translational repression module. Future work will need to address if transcriptional and translational repression is a conserved control mechanism of regulation of nodals and nodal pathway components across species. Indeed, experiments in *Xenopus* suggest that translation of the Nodal co-receptor Cripto is spatially regulated in the early frog embryo [92,94] and dampening of Nodal signalling in the human placenta by miRNA-378a-5p is required for trophoblast proliferation and invasion. In addition, a recent report shows that translational repression of Lefty by miRNA-430 regulates the dimensions of Nodal signalling and mesendoderm specification during zebrafish gastrulation [103].

Nodal signalling also regulates cell-fate decisions in hESCs. Inhibition of nodal signalling in hESCs leads to formation of extravillous trophoblast cells, whereas loss of activin/nodal inhibition (i.e. gain-of-nodal signalling) leads to the formation of syncytiotrophoblasts [104,105]. Given that the left asymmetric enhancer, FoxH1, Smad2/3 and miR430-mediated regulation are shared by *nodal* and *lefty* genes across many species, it seems likely that in embryonic progenitor cells, Nodal and other components of the Nodal pathway might be tightly regulated by transcriptional as well as translational repression.
